# Good-Bye—The Editor Retires

**Published:** 1877-11

**Authors:** 


### "GOOD BYE—THE EDITOR RETIRES."

"For the last two or three years I have advocated practices that many dentists disapprove; I have made personal enemies by advocating principles of practice, of ethics, and of dental education; I have had a very good opportunity to put myself in print. It is time that I retire. Some one else may do better for the profession than I have. I have not worked for the profession alone. I have had the good of the public in view also; I am not popular; I voluntarily retire."

HENRY S. CHASE.

The above from the Missouri Dental Journal, indicates pretty clearly the withdrawal of Dr. Chase from the editorial tripod. There will be many regrets for this, notwithstanding the Dr's "unpopularity." We doubt if anybody would have found this out if the Dr. had not told it himself. Dr. S. has the reputation of being an industrious and earnest worker. He has often taken position with which very many did not agree, but then he should not have become discouraged because of that; he has escaped criticism wonderfully well, when we take into account the ways in which he has so frequently exposed himself.

His writings would rather indicate that when an idea strikes him, it is with the large end first, and that it so impresses him that he must out with it. A little patient consideration would, sometimes at least, have saved him from that of which he so sorely complains. But Dr. C. is, in many respects, a good writer and we trust the profession will not wholly lose his efforts in this direction.—The above was written two months ago, but has been crouded out of place until now.

---

Premium for 1878.—We will announce in next issue, the Premium for each subscription to Dental Register for 1878.

Spencer & Crocker.

